# *Epinephelus tankahkeei*, a new species of grouper (Teleostei, Perciformes, Epinephelidae) from the South China Sea

**DOI:** 10.3897/zookeys.933.46406

**Published:** 2020-05-18

**Authors:** Haohao Wu, Meng Qu, Hungdu Lin, Wei Tang, Shaoxiong Ding

**Affiliations:** 1 Marine Biodiversity and Global Change Research Center, College of Ocean and Earth Science, Xiamen University, Xiamen 361102, China Xiamen University Xiamen China; 2 The Affiliated School of National Tainan First Senior High School, Tainan, Taiwan The Affiliated School of National Tainan First Senior High School Tainan Taiwan; 3 Function Laboratory for Marine Fisheries Science and Food Production Processes, Qingdao National Laboratory for Marine Science and Technology, Qingdao 266200, China Qingdao National Laboratory for Marine Science and Technology Qingdao China

**Keywords:** *
Epinephelidae
*, *Epinephelus tankahkeei*, groupers, new species, South China Sea

## Abstract

A new species of grouper, *Epinephelus tankahkeei***sp. nov.** is described from the South China Sea based on examination of morphological and molecular characteristics. This new species has been treated as, and is similar to, its congener *E. chlorostigma*. *Epinephelus tankahkeei***sp. nov.** can be distinguished from *E. chlorostigma* by the following combination of characters: a convex anal fin; closer dark spots on the body; a lack of dark spots on the abdomen, cheek, and pectoral fin; the absence of a clear posterior white margin on the caudal fin. Molecular analyses of the mitochondrial *COI* sequence variation, genetic distances, and a phylogeny, all highly support *E. tankahkeei***sp. nov.** as a distinct species. A key to *E. tankahkeei***sp. nov.** and its most closely related species is provided.

## Introduction

The groupers are an assemblage of reef fishes in the perciform family Epinephelidae (Smith and Craig 2007; Craig et al. 2011; Zhuang et al. 2013), comprising more than 160 species in 16 genera (Heemstra and Randall 1993; Craig et al. 2011). The genus *Epinephelus* Bloch, 1793 (type species: *Epinephelus marginalis* Bloch, 1793 = *Epinephelus fasciatus*) is the most biologically diverse of all grouper genera (Heemstra and Randall 1993) and contains more than 90 valid species (Frable et al. 2018). These species are characterized by an elongate, robust (subcylindrical), oblong or deep and compressed body; a dorsal fin usually with XI spines (X spines in some species) and 12 to 19 rays; and an anal fin with III distinct spines and 7 to 10 (very rarely 7 or 10) rays. *Epinephelus* spp. are widespread in the rocky and reef shores of tropical and subtropical oceans, and are usually apex predators in their habitats. They are also commercially important and constitute a significant component of coastal fisheries (Dalzell et al. 1996). Due to the ecological and economic importance of these species, their alpha taxonomy and phylogenetic relationships have been well reviewed (Craig and Hastings 2007; Ma et al. 2016). However, groupers appear to have undergone rapid sympatric speciation and usually show fewer differences in morphology between closely related species, thus some cryptic species might still be undiscovered. Therefore, the use of genetic data is of considerable importance in grouper taxonomic and diversity research (Gilles et al. 2000; Han et al. 2011).

In recent years, we collected a new form of grouper from the South China Sea that had been previously regarded as *Epinephelus chlorostigma*. Further investigation based on morphometric and molecular characteristics shows that this new form should be a new species of the genus *Epinephelus*. Herein, we describe this new species as *Epinephelus tankahkeei*. In addition, a key to *E. tankahkeei* sp. nov. and its most closely related species is provided.

## Materials and methods

Between 2011 and 2019, nine specimens of the new species were collected from fish markets and fishing boats in Xiamen, Shenzhen, Sansha, and Haikou, China. ODV v5.1.5 software was used to generate a collection site map (Schlitzer 2002). The sampling localities are listed in Suppl. material 1: Table S1. The holotype and paratypes were fixed and preserved in anhydrous ethanol. The specimens were stored in the Fish Collection of the College of Ocean and Earth Sciences, Xiamen University. Institutional codes followed Sabaj (2016).

The methods of counting and measurement followed Randall and Heemstra (1993) and include: total length; standard length (as SL); head length; snout length; body depth; body width; orbit diameter; interorbital width; preorbital depth; maxilla width; upper jaw length; lower jaw length; length of pelvic-fin and anal-fin spines; lengths of the dorsal, anal, pectoral, pelvic and caudal fins; caudal-peduncle depth; caudal-peduncle length; predorsal length; preanal length; prepelvic length; dorsal-fin base; longest hard dorsal spine; longest soft dorsal ray; anal-fin base; and length of the third anal spine, longest anal soft ray, and pelvic-fin spine. The following counts were made: gill rakers, lateral-line scales, lateral scale series, pectoral-fin rays, anal-fin rays, dorsal-fin rays, pelvic-fin rays, caudal-fin rays, and vertebras.

The procedures for DNA isolation, PCR amplification and sequencing followed Qu et al. (2018). DNA was extracted using the standard phenol-chloroform protocol and the ethanol precipitation method and then stored at -20 °C. Polymerase chain reaction (PCR) was performed to amplify the partial fragment of the mitochondrial *COI* locus using a pair of primers (Fish F1, 5’-TCAACCAACCACAAAGACATTGGCAC-3’ and Fish R1, 5’-TAGACTTCTGGGTGGCCAAAGAATCA-3’) (Ward et al. 2005). The thermal cycler program for PCR was 95 °C for 5 min, followed by 35 cycles of 94 °C for 30 s, 52 °C for 30 s and 72 °C for 45 s and a final extension at 72 °C for 10 min. The products were checked by electrophoresis on a 1% agarose gel to confirm the predicted fragment size and were then sequenced. The sequencing results were trimmed and manually proofread using SEQUENCHER 5.4.6 (http://www.genecodes.com) software. All sequences in this study were deposited in GenBank, and the accession numbers are shown in Suppl. material 1: Table S1.

Due to the availability of data for other related species in GenBank, we chose the mitochondrial *COI* gene sequence to calculate intraspecific and interspecific genetic distances and perform maximum likelihood (ML) and Bayesian analyses in this study. The intraspecific and interspecific genetic distances were generated using the Kimura two-parameter (K2P) distance model with MEGA 7 (Kumar et al. 2016). For the phylogenetic analyses, *Epinephelus akaara* (GenBank No. MF185437) and *Epinephelus awoara* (GenBank No. MF185456) were used as the outgroups because they are located in a clade sister to the one containing the *E. chlorostigma* species-complex (Ma et al. 2016). jModelTest 2.1.9 was used to infer the best evolutionary model, and the TrN+I+G model was selected based on both the Akaike information criterion (AIC) and the Bayesian information criterion (BIC) (Darriba et al. 2012). ML phylogenetic analysis was performed with the PhyML 3.1 program with 1000 bootstrap replicates (Guindon and Gascuel 2003), and the Bayesian phylogenetic analysis was performed by using MrBayes 3.2.6 (Ronquist et al. 2012).

## Taxonomy

### Epinephelus tankahkeei sp. nov.

Taxon classificationAnimalia

ACDF350B-704B-56DE-9323-AA7CDAB0C62E

http://zoobank.org/1C62B8C1-33B3-4A75-88D9-C49B5897EA55

**Figure 1.**
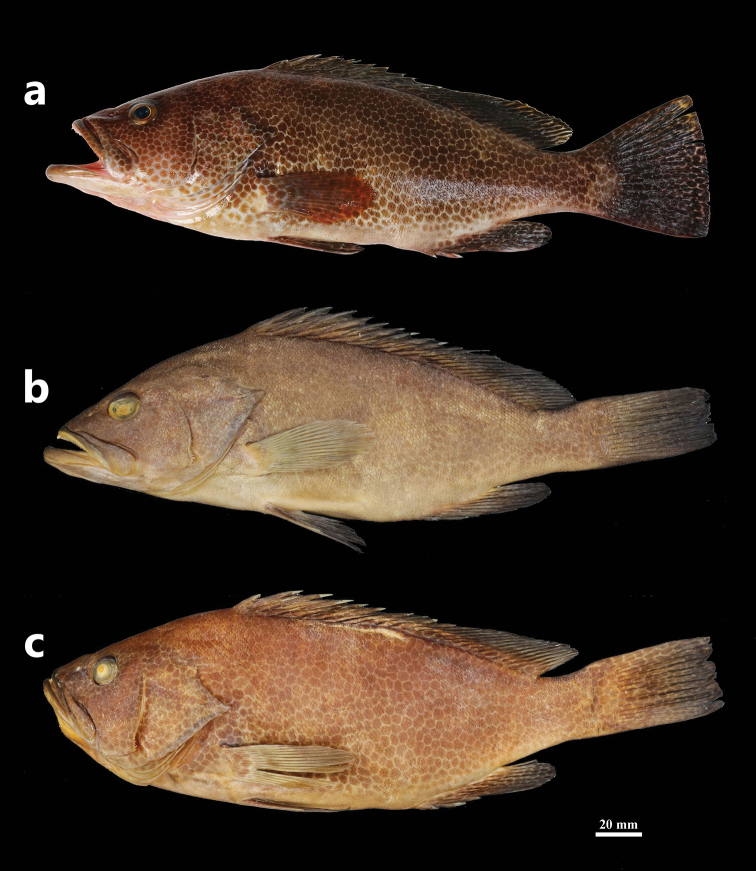
*Epinephelus tankahkeei***a** holotype, ZMUA-eptan06, 244.5 mm SL, Xisha Islands from the South China Sea **b** preserved holotype **c** paratype, ZMUA-eptan02, 233.2 mm SL, purchased at the Bashi market in Xiamen, Fujian, China, 22 August 2016.

**Figure 2.**
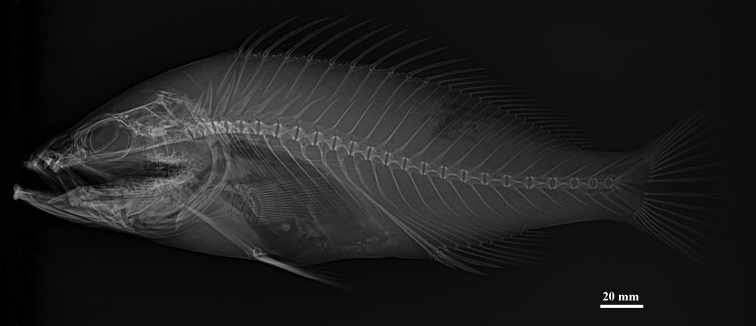
*Epinephelus tankahkeei* sp. nov. Radiograph of paratype ZMUA-eptan08, 274.2 mm SL.

Figs 1 , 2 ; Table 1

#### Type material.

***Holotype***: ZMUA-eptan06, 244.5 mm SL, Caught in Yongxing Island, Sansha, Hainan, 2 April 2017. ***Paratypes***: ZMUA-eptan01, 111.0 mm SL, China, purchased at a fish market in Xiamen, Fujian, China, 1 September 2011, reported as collected in the south Taiwan Strait; ZMUA-eptan02, 233.2 mm SL, purchased at the Bashi market in Xiamen, Fujian, China, 22 August 2016, reported as collected in the south Taiwan Strait; ZMUA-eptan03, 215.4 mm SL, collected with the ZMUA-eptan02; ZMUA-eptan04, 262.5 mm SL, purchased at a fish market in Shenzhen, Guangdong, China, 15 February 2017, reported as collected in the South China Sea; ZMUA-eptan05, 232.6 mm SL, collected with the holotype; ZMUA-eptan07, 252.9 mm SL, purchased at a fish market in Shenzhen, Guangdong, China, 1 July 2017, reported as collected in the South China Sea; ZMUA-eptan08, 274.2 mm SL, China, purchased at a fish market in Xiamen, Fujian, China, 31 July 2018, reported as collected in the south Taiwan Strait. ZMUA-eptan09, 186.5 mm SL, China, purchased at a fish market in Haikou, Hainan, China, 22 March 2019, reported as captured using a trawl net in Mulan Bay, Wenchang, Hainan.

#### Diagnosis.

*Epinephelus tankahkeei* sp. nov. can be distinguished from all other Indo-Pacific *Epinephelus* species by the following characteristics: dorsal-fin rays XI, 16–18 (vs. 14–15 in *Epinephelus gabriellae*); anal-fin rays III, 8; pectoral-fin rays 16 or 17; lateral-line scales 47–51 (vs. 65–72 in *Epinephelus polylepis*); caudal fin convex (vs. slightly emarginate or truncate caudal fin in *E. chlorostigma*, *Epinephelus areolatus*, *Epinephelus bleekeri*, and *Epinephelus geoffroyi*) ; anal fin rounded (vs. angular anal fin in *E. chlorostigma*, and *E. geoffroyi*); membranes of spinous portion of dorsal fin slightly incised; head (except chest), body (except abdomen), and fins (pectoral fin spotted only basally) with numerous, irregular, close-set, dark brown spots, becoming more widely spaced on the lower part, the ground color forming a pale network (vs. lager spots in *Epinephelus miliaris*, and *E. areolatus*); rear margin of the caudal fin without a narrow white line (vs. a clear white margin posteriorly on the caudal fin in *E. chlorostigma*, and *E. areolatus*).

#### Description.

Dorsal-fin rays XI, 16 (16–18); anal-fin rays III, 8; pectoral-fin rays 16 (16 or 17); lateral-line scales 51 (47–51); lateral scale series 123 (111–123); gill rakers 10 (10–11) +15 (14–16); vertebra 24; body slightly elongated and body depth less than head length. Body depth contained 3.2 (3.0–3.2) in standard length. Body laterally compressed and body width 2.6 (2.1–2.6) in body depth. Head length 2.7 (2.5–2.7) in SL; orbit diameter 6.1 (4.9–6.8) in head; snout length 3.8 (3.6–4.0) in head. Dorsal profile of head nearly straight except at the snout with a slight notch before eyes. Interorbital region convex, width 6.3 (5.6– 7.2) in head; preorbital depth 7.8 (7.8–11.3) in head; caudal-peduncle length 1.9 (1.8–2.2) in head; caudal-peduncle depth 3.3 (3.2–3.7).

Mouth large and lower jaw slightly projecting and oblique. Lower jaw 3.4 (3.4–3.9) in head length; upper jaw 2.4 (2.3–2.5) in head. Maxilla slightly extending to rear edge of eye and posterior edge of maxilla slightly rounded. Maxilla width 8.1 (7.9–9.2) in head. One or two pairs of canine teeth at anterior part of the upper and lower jaw. Teeth of lower jaw form two rows and expand anteriorly into three rows; teeth in the outer side are larger than the inner side. Villiform teeth present on vomer and palatines. Tongue slender and sharp at tip. Longest gill raker was greater in length than longest gill filament. Nostrils round and posterior nostril larger than anterior nostril. Anterior nostril with a membranous flap.

Three spines on operculum, topmost and undermost small, the middle the largest. Tip of middle spine extending farther towards tail than tip of lower spine. Upper edge of opercular membrane slightly convex coming to a rounded point posteriorly. Preopercle rounded with four to five prominent spines at angle and with numerous fine serrae while increasing in size downward. Lateral line starting from posterior opercle and slightly arched over pectoral region. Scales on head, thorax, abdomen, anterodorsal part of body and fin membranes weakly ctenoid. Auxiliary scales absent. Small scales present on inner margins of dorsal, pectoral, pelvic, and caudal fins and not extending to the rear margin area.

Origin of dorsal fin before pectoral-fin base. Membranes of spinous portion of dorsal fin slightly incised. First dorsal spine contained 1.9 (1.6–2.0) times in second spine; second spine 1.2 (1.2–1.5) times in longest spine (third and fourth spine); longest spine contained 2.6 (2.6–3.1) in head length. Longest soft dorsal ray 2.4 (2.3–2.8) in head. Anal-fin origin below origin of first soft dorsal ray. First anal spine 2.0 (1.9– 2.3) times in second anal spine; second anal spine 1.2 (1.1– 1.4) times in third anal spine; third anal spine longest 3.5 (3.0–4.1) in head. Longest anal-fin rays 2.1 (1.9–2.5) in head. Caudal fin convex, 1.7 (1.6–2.0) in head. Middle pectoral rays longest, 1.8 (1.7–1.9) in head and reaching to base of 9^th^ dorsal spine. Origin of pelvic fin slightly posterior to pectoral-fin base.

**Table 1.** Meristics and measurements for type specimens of *Epinephelus tankahkeei* and *E. chlorostigma*. The dashes indicate that data were not collected due to specimen damage, which prevented an accurate measurement or count.

	***Epinephelus tankahkeei***		***E. chlorostigma***	
	**ZMUA-eptan06 (holotype)**	**Range for all type specimens**	**ANSP 103722**	**ANSP 163245**
Standard length (mm)	244.5	111–274.2	281	398
Total length (mm)	286.2	136.5–334.7	355	503
Dorsal-fin ray count	XI, 17	XI, 16–18	XI, 16	XI, 16
Anal-fin ray count	III,8	III,8	III,8	III,8
Pectoral-fin ray count	16	16–17	18	17
Pelvic-fin ray count	I+5	I+5	I+5	I+5
Lateral line scales	51	47–51	52	58
Lateral scale series	123	111–123	102	106
Gill rakers count	10+15	10–11+14–16	9+14	8+18
% of SL				
Body depth	31.1	31.1–33.7	34.9	35.6
Body width	12.2	12.2–15.6	16.3	18.1
Head length	37.1	36.9–39.6	38.7	39.2
Snout length	9.8	9.5–10.9	10.0	10.1
Orbit diameter	6.1	5.7–8	7.5	6.1
Preorbital depth	4.8	3.5–4.8	4.7	4.5
Interorbital width	5.8	5.5–6.7	7.2	8
Maxilla width	4.6	4.1–4.8	4.9	5.1
Upper jaw length	15.6	15.6–17.1	18.0	17.5
Lower jaw length	11	8.6–11.5	12.4	11.7
Caudal peduncle depth	11.1	10.5–12.1	11.9	10.8
Caudal peduncle length	19.2	18–21.1	20.0	22.7
Predorsal length	34.6	32.4–38.8	36.4	36.5
Preanal length	63.4	58.5–69.1	68.7	73.9
Prepelvic length	36.8	34.8–40.8	38.8	34.7
Dorsal-fin base	53.4	53.1–61.4	59.4	54.8
Longest hard dorsal ray	14.3	12.4–14.4	15.7	14.3
Longest soft dorsal ray	15.2	13.4–16.9	14.5	15.4
Anal-fin base	15	14.8–17.3	15.4	16.2
Third anal spine length	10.7	9.3–13.3	10.0	8.1
Longest anal soft ray	17.3	14.9–21.1	15.7	15
Caudal-fin length	22.1	18.7–24.2	25.5	22.3
Pectoral-fin length	20.1	19.6–23.2	20.9	20.2
Pelvic-fin length	19.5	18.1–21.7	20.4	17.4
Pelvic spine length	11	9.7–12.6	10.4	9.2

#### Coloration in life

**(based on photographs of the fresh holotype and paratypes).** Head (except chest), body (except abdomen), and fins (pectoral fin only basally) with numerous, irregular, close-set, dark brown spots becoming more widely spaced on the lower part and with the ground color forming a pale network (Fig. 1a); dorsal fin, caudal fin and anal fin dark brown; pectoral fin translucent with reddish brown to light yellowish-brown; body sometimes with four faint, irregular, discrete dark bars; rear margin of the caudal fin without a narrow white line.

#### Coloration in preservative.

Body yellowish-brown to tan with close-set spots remaining prominent or faded (Fig. 1b, c). Dorsal, caudal, and anal fins dark brown. Pectoral fin pale and opaque.

#### Genetic analyses.

**Figure 3.**
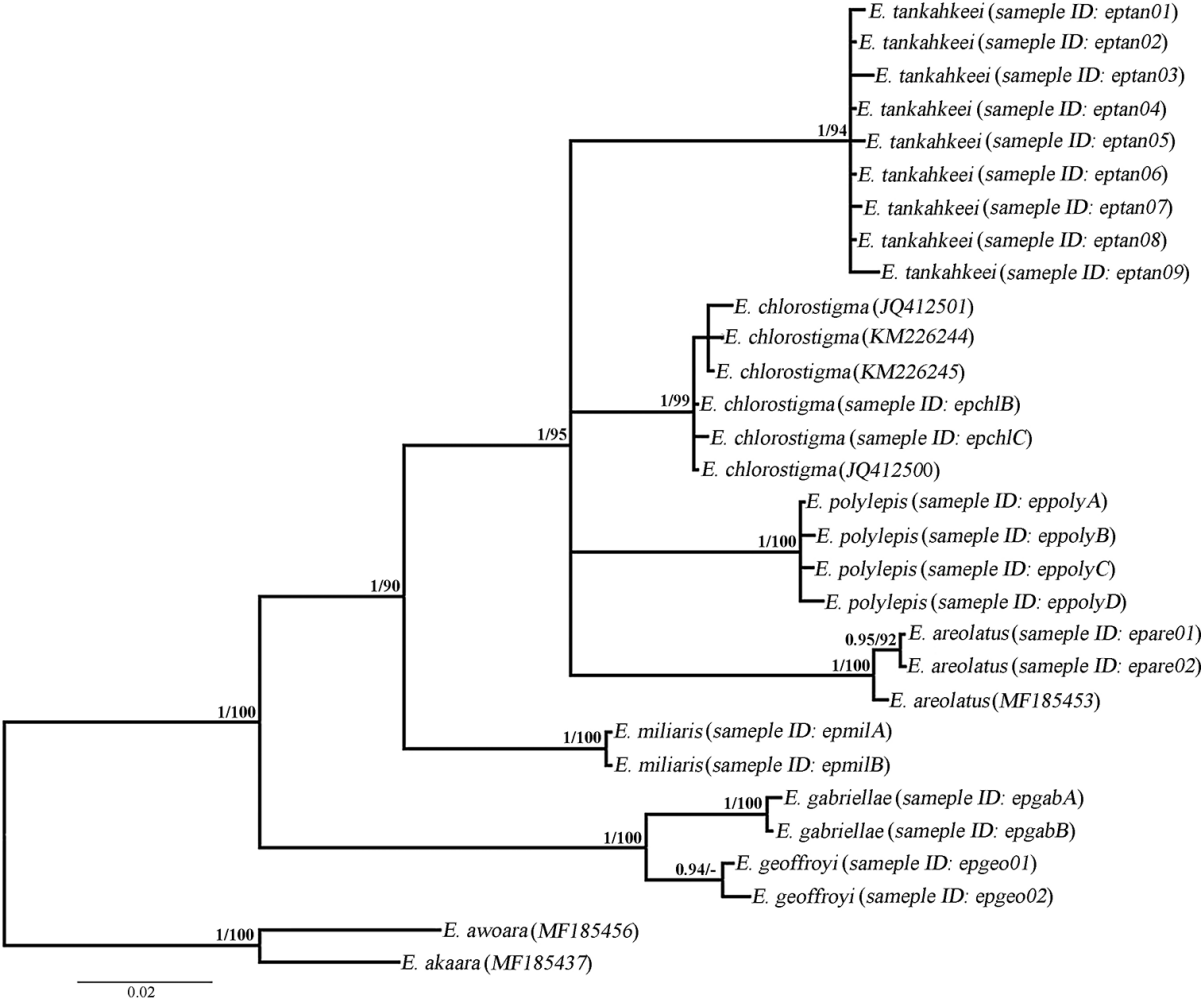
Bayesian phylogenetic tree of *Epinephelus tankahkeei* and closely related fish species. Numbers above nodes are Bayesian posterior probability values (left) and ML bootstrap values above 50 (right).

Mitochondrial *COI* gene sequences were obtained from nine specimens of *E. tankahkeei*. Several sequences of related species were also sequenced in this study or obtained from GenBank. *E. tankahkeei* has 13 species-specific mutations at nucleotide positions 126, 216, 222, 249, 276, 372, 414, 519, 525, 528, 558, 567, and 576 (Table 2). The intraspecific mean distance of *E. tankahkeei* was 0.0028. The interspecific mean distances indicated that *E. tankahkeei* differs from *E. chlorostigma* by 0.0621, from *E. polylepis* by 0.0771, from *E. gabriellae* by 0.1263, from *E. miliaris* by 0.0904, from *E. geoffroyi* by 0.1219, and from *E. areolatus* by 0.0855 (Table 3). Phylogenetic trees using both maximum likelihood and Bayesian inference showed almost complete agreement, with *E. tankahkeei* forming a monophyletic clade that excluded all other closely related species (Fig. 3).

#### Distribution and habitat.

The new species was recently observed in the South China Sea and Taiwan Strait. Similar to other congeners, *E. tankahkeei* is a reef-associated species that feeds on fishes and invertebrates.

#### Etymology.

*Epinephelus tankahkeei* is named after Tan Kah Kee (1874–1961), who was a famous overseas Chinese educator, philanthropist, and social activist and the founder of Xiamen University and Jimei School, in honor of his significant contribution to the motherland.

**Table 2.** Species-specific mutation sites in the *COI* gene for *Epinephelus tankahkeei*.

	Nucleotide position (beginning from 5’ end)												
	126	216	222	249	276	372	414	519	525	528	558	567	576
*Epinephelus tankahkeei*	T	C	C	A	C	G	G	T	G	C	T	G	C
Other closely related species in this study	C	A, G	T	T, C	A	A	A	A, G	C	G, A	A, G, C	A	A, T

**Table 3.** Analysis of the intraspecific and interspecific (K2P model) distances, interspecific distances (in lower left) and standard errors (in upper right) based on the *COI* locus between *Epinephelus tankahkeei* and closely related species; IMD = Intraspecific mean distance; SE = standard error.

					Interspecific Mean Distance						
Group	Species	*N*	IMD	SE	1	2	3	4	5	6	7
1	*Epinephelus tankahkeei*	9	0.0028	0.0010	–	0.0112	0.0100	0.0144	0.0123	0.0141	0.0122
2	*E. polylepis*	4	0.0031	0.0015	0.0771	–	0.0093	0.0144	0.0127	0.0136	0.0119
3	*E. chlorostigma*	6	0.0030	0.0013	0.0621	0.0545	–	0.0143	0.0110	0.0138	0.0110
4	*E. gabriellae*	2	0.0016	0.0015	0.1263	0.1142	0.1169	–	0.0148	0.0066	0.0154
5	*E. miliaris*	2	0.0000	0.0000	0.0904	0.0885	0.0730	0.1143	–	0.0143	0.0122
6	*E. geoffroyi*	2	0.0047	0.0026	0.1219	0.1073	0.1107	0.0321	0.1107	–	0.0143
7	*E. areolatus*	3	0.0031	0.0018	0.0855	0.0831	0.0641	0.1239	0.0918	0.1152	–

## Discussion

*Epinephelus chlorostigma* was formerly reported to have a wide distribution range from the Red Sea and the coast of Africa to the western Pacific Ocean. It was considered a species complex (the *E. chlorostigma* species complex) (Heemstra and Randall 1993). Since the early 1990s, new species have been successively distinguished from *E. chlorostigma* and described. *Epinephelus gabriellae* has a restricted range from Oman to Somalia and differs in having fewer dorsal-fin rays (14–15 vs. 16–18). *Epinephelus polylepis* is distributed from the western coast of India to the coast of Yemen and has more lateral-line scales and lateral-scale series (65–72 and 126–137 vs. 48–53 and 96–122, respectively) (Randall and Heemstra 1991). *Epinephelus geoffroyi* is local to the Red Sea and has more gill rakers (25–29 vs. 23–26) (Randall et al. 2013). Interestingly, the three recently described species above are all distributed to the west of the Indo-Australian Archipelago (IAA), even though the type locality of *E. chlorostigma* is the Seychelle Islands in the Indian Ocean. Currently, *E. tankahkeei* collected from the China Seas can be morphologically distinguished from *E. chlorostigma* by its rounder anal fin, closer dark spots on the body, lack of dark spots on the abdomen, and lack of a narrow, pale whitish posterior margin on the caudal fin.

Our molecular analyses also corroborated the morphological results. In *E. tankahkeei*, 13 species-specific mutations were found in the *COI* gene fragment (Table 2). Genetic distance analysis also revealed high divergence between *E. tankahkeei* and its closely related species. The interspecific mean distance between *E. tankahkeei* and *E. chlorostigma* was 0.0621, which was greater than the distance (0.0545) between *E. chlorostigma* and *E. polylepis* (Table 3). The phylogenetic analyses performed with both ML and Bayesian inference also revealed a distinct monophyletic group formed by all samples of *E. tankahkeei*, and this group was separated from *E. chlorostigma*, *E. polylepis*, and *E. areolatus*. Although the phylogenetic relationships of the four species could not be well resolved by only the *COI* gene, each of the species formed a monophyletic clade with high support, supporting their validity (the ML bootstrap value was 94% for *E. tankahkeei*, 99% for *E. chlorostigma*, 100% for *E. polylepis*, and 100% for *E. areolatus*) (Fig. 3).

In the China Seas, the first record of *E. chlorostigma* was *Serranus reevesii* Richardson, 1846, type locality Canton, China (based on a painting by John Reeves) (Richardson 1846), which was later treated as a synonym of *E. chlorostigma* (Heemstra and Randall 1993). However, it is difficult to confirm the validity of *S. reevesii* due to its unclear description and lack of reliable photos and type specimen. As mentioned by Randall and Heemstra (1991), there are no confirmed records of *E. chlorostigma* in the continental waters of Asia. Our sampling in the China Seas over the last 20 years also never resulted in any *E. chlorostigma* specimens. Together with the distribution range, morphological characteristics, and molecular data, we suppose that most, or even all, of the former records of *E. chlorostigma* in the China Seas might be misidentifications of *E. tankahkeei*. More samples should be taken in the future to verify the distribution range of *E. chlorostigma*.

**Figure 4.**
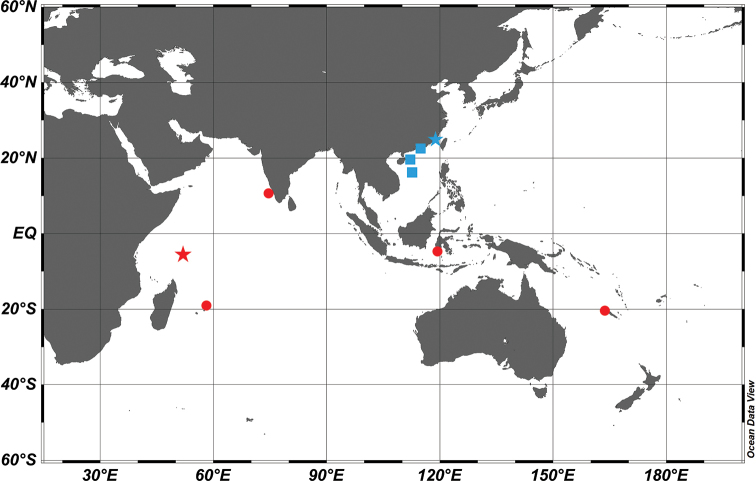
A map of the collection sites of *Epinephelus tankahkeei* (blue squares) and *E. chlorostigma* (red circles) examined in this study. The blue star represents the collection site of the *E. tankahkeei* holotype (ZMUA-eptan06), and the red star represents the type locality of *E. chlorostigma*.

### Key to *Epinephelus tankahkeei* and closely related species

**Table:** 

1a	Lateral-line scales 65–72, lateral-scale series 126–137	***E. polylepis***
1b	Lateral-line scales 47–54, lateral-scale series 92–126	**2**
2a	Dorsal-fin rays 14 or 15; body depth 3.2 to 3.6 times in standard length [coast of Oman, eastern border of Yemen, Somalia]	***E. gabriellae***
2b	Dorsal-fin rays 16 to 18 (rarely 15 in *E. areolatus*); body depth 2.8 to 3.3 times in standard length	**3**
3a	Caudal fin rounded or convex	**4**
3b	Caudal fin slightly emarginate (truncate on some specimens of *E. chlorostigma*)	**5**
4a	Body and fins with dark brown to black spots, those spots on fins (except spinous dorsal fin) much larger than those on body; lateral-scale series 92 to 108 [Indo-Pacific but not in Red Sea and Persian Gulf]	***E. miliaris***
4b	Body and fins with dark brown spots, becoming more widely spaced on the lower part; lateral-scale series 114 to 123 [South China Sea and Taiwan Strait]	***E. tankahkeei***
5a	Dorsal-fin rays 15 to 17; anal fin margin in adults rounded or slightly angular; dark spots on body of adults subequal to pupil [Red Sea to western Pacific]	***E. areolatus***
5b	Dorsal-fin rays 16 to 18; anal fin of adults angular; largest dark spots on body of adults distinctly smaller than pupil	**6**
6a	Posterior margin of caudal fin without a narrow, clear whitish margin; margin of anal fin distinctly angular; gill rakers 25–29 [Red Sea]	***E. geoffroyi***
6b	Posterior margin of caudal fin with a narrow, clear whitish margin; margin of anal fin slightly angular; gill rakers 23–26 [Western Indian Ocean to western Pacific]	***E. chlorostigma***

### Comparative material examined

*Epinephelus areolatus*: ANSP 54826, ZMUA-epare01, ZMUA-epare02, and ZMUA-epare03.

*Epinephelus chlorostigma*: FLMNH_I 2006-0681, FLMNH_I 2007-1089, ANSP 162821, ANSP 163245, and ANSP 103722.

*Epinephelus miliaris*: FLMNH_I 2006-0784 and FLMNH_I 2006-0728.

*Epinephelus polylepis*: FLMNH_I 2005-1073.

*Epinephelus geoffroyi*: ZMUA-epgeo01 and ZMUA-epgeo02.

(See Suppl. material 1: Table S1 for more information.)

## Supplementary Material

XML Treatment for Epinephelus tankahkeei

## Supplementary materials

Supplementary material 1 Table S1. Samples information Data type samples data https://binary.pensoft.net/file/411313This dataset is made available under the Open Database License (http://opendatacommons.org/licenses/odbl/1.0/). The Open Database License (ODbL) is a license agreement intended to allow users to freely share, modify, and use this Dataset while maintaining this same freedom for others, provided that the original source and author(s) are credited.Haohao Wu, Meng Qu, Hungdu Lin, Wei Tang, Shaoxiong Ding

## References

[B1] Craig MT Hastings PA (2007) A molecular phylogeny of the groupers of the subfamily Epinephelinae (Serranidae) with a revised classification of the Epinephelini. Ichthyological Research 54: 1–17. 10.1007/s10228-006-0367-x

[B2] Craig MT Sadovy de Mitcheson YJ Heemstra PC (2011) Groupers of the world: A Field and Market Guide. NISC (Pty) Ltd, Grahamstown, 356 pp.

[B3] Dalzell PJ Adams TJH Polunin NVC (1996) Coastal fisheries in the Pacific islands. Oceanography and Marine Biology: 395–531. http://www.spc.int/DigitalLibrary/Doc/FAME/Reports/Dalzell_96_OMB.pdf

[B4] Darriba D Taboada GL Doallo R Posada D (2012) jModelTest 2: more models, new heuristics and parallel computing. Nature methods 9: 1–772. 10.1038/nmeth.2109 22847109 PMC4594756

[B5] Frable BW Tucker SJ Walker HJ (2018) A new species of grouper, Epinephelus craigi (Perciformes: Epinephelidae), from the South China Sea. Ichthyological Research: 1–10. 10.1007/s10228-018-0669-9

[B6] Gilles A Miquelis A Quignard JP Faure É (2000) Molecular phylogeograpby of western Mediterranean dusky grouper Epinephelus marginatus. Comptes Rendus de l’Academie des Sciences - Serie III 323: 195–205. 10.1016/S0764-4469(00)00114-1 10763438

[B7] Guindon S Gascuel O (2003) A simple, fast, and accurate algorithm to estimate large phylogenies by maximum likelihood. Systematic biology 52: 696–704. 10.1080/10635150390235520 14530136

[B8] Han J Lv F Cai H (2011) Detection of species-specific long VNTRs in mitochondrial control region and their application to identifying sympatric Hong Kong grouper (Epinephelus akaara) and yellow grouper (Epinephelus awoara). Molecular Ecology Resources 11: 215–218. 10.1111/j.1755-0998.2010.02911.x 21429126

[B9] Heemstra PC Randall JE (1993) FAO Species Catalog (Vol. 16). Groupers of the World (Family Serranidae, Subfamily Epinephelinae) An Annotated and Illustrated Catalogue of the Grouper, Rockcod, Hind, Coral Grouper and Lyretail Species Known to Date. FAO Fisheries Synopsis No. 125. FAO, Rome, 382 pp.

[B10] Kimura M (1980) A simple method for estimating evolutionary rates of base substitutions through comparative studies of nucleotide sequences. Journal of Molecular Evolution 16: 111–120. 10.1007/BF01731581 7463489

[B11] Kumar S Stecher G Tamura K (2016) MEGA7: molecular evolutionary genetics analysis version 7.0 for bigger datasets. Molecular biology and evolution 33: 1870–1874. 10.1093/molbev/msw054 27004904 PMC8210823

[B12] Ma KY Craig MT (2018) An Inconvenient Monophyly: An Update on the Taxonomy of the Groupers (Epinephelidae). Copeia 106: 443–456. 10.1643/CI-18-055

[B13] Ma KY Craig MT Choat JH van Herwerden L (2016) The historical biogeography of groupers: Clade diversification patterns and processes. Molecular Phylogenetics and Evolution 100: 21–30. 10.1016/j.ympev.2016.02.012 26908372

[B14] Qu M Tang W Liu Q Wang D Ding S (2018) Genetic diversity within grouper species and a method for c hybrid identification using DNA barcoding and RYR3 marker. Molecular Phylogenetics and Evolution 121: 46–51. 10.1016/j.ympev.2017.12.031 29294404

[B15] Randall JE Heemstra PC (1991) Revision of Indo-Pacific groupers (Perciformes: Serranidae: Epinephelinae), with descriptions of five new species. Indo-Pacific Fishes 20: 1–322.

[B16] Randall JE Bogorodsky SV Krupp F Rose JM Fricke R (2013) Epinephelus geoffroyi (Klunzinger, 1870) (Pisces: Serranidae), a valid species of grouper endemic to the Red Sea and Gulf of Aden. Zootaxa 3641: 524–532. 10.11646/zootaxa.3641.5.2 26287104

[B17] Richardson J (1846) Report on the ichthyology of the seas of China and Japan. Report of the British Association for the Advancement of Science 15^th^ meeting (1845): 187–320. 10.5962/bhl.title.59530

[B18] Ronquist F Teslenko M Van Der Mark P Ayres DL Darling A Höhna S Larget B Liu L Suchard MA Huelsenbeck JP (2012) Mrbayes 3.2: Efficient bayesian phylogenetic inference and model choice across a large model space. Systematic Biology 61: 539–542. 10.1093/sysbio/sys029 22357727 PMC3329765

[B19] Sabaj MH (2016) Standard Symbolic Codes for Institutional Resource Collections in Herpetology and Ichthyology: an Online Reference. Version 6.5 (16 August 2016). http://www.asih.org/ resources/standard-symbolic-codes-institutionalresource-collections-herpetology-ichthyology

[B20] Schlitzer R (2002) Interactive analysis and visualization of geoscience data with Ocean Data View. Computers & geosciences 28: 1211–1218. 10.1016/S0098-3004(02)00040-7

[B21] Smith WL Craig MT (2007) Casting the Percomorph Net Widely: The Importance of Broad Taxonomic Sampling in the Search for the Placement of Serranid and Percid Fishes. Copeia 2007: 35–55. 10.1643/0045-8511(2007)7[35:CTPNWT]2.0.CO;2

[B22] Ward RD Zemlak TS Innes BH Last PR Hebert PDN (2005) DNA barcoding Australia’s fish species. Philosophical Transactions of the Royal Society B: Biological Sciences 360: 1847–1857. 10.1098/rstb.2005.1716 PMC1609232 16214743

[B23] Zhuang X Qu M Zhang X Ding S (2013) A Comprehensive Description and Evolutionary Analysis of 22 Grouper (Perciformes, Epinephelidae) Mitochondrial Genomes with Emphasis on Two Novel Genome Organizations. PLoS ONE 8: 1–14. 10.1371/journal.pone.0073561 PMC3739747 23951357

