# Supplementary material for: Epinephelus tankahkeei, a new species of grouper (Teleostei, Perciformes, Epinephelidae) from the South China Sea
Source: Zookeys. 2020 May 18;933:125–37. doi: 10.3897/zookeys.933.46406 (PMC7248128; doi:10.3897/zookeys.933.46406)
Supplement: Supplementary material 1 — Table S1. Samples information [file zookeys-933-125-s001.docx]

**Supplementary Table 1.** Samples information

| Species | Individual Number | Samples ID | Collection Date | Collection Locality | GenBank Accession Numbers | References |
| --- | --- | --- | --- | --- | --- | --- |
|  |  |  |  |  | *COI* |  |
| *Epinephelus akaara* | 1 | epaka01 | Feb-2011 | Xiamen, Fujian | MF185437 | Qu et al. 2018 |
| *E. awoara* | 1 | epawoA | Apr-2006 | Xiamen, Fujian | MF185456 | Qu et al. 2018 |
| *E. areolatus* | 3 | epare01 | Nov-2011 | Sanya, Hainan | MF185454 | Qu et al. 2018 |
|  |  | epare02 | Mar-2011 | Xiamen, Fujian | MF185455 | Qu et al. 2018 |
|  |  | epareA | Apr-2005 | Xiamen, Fujian | MF185453 | Qu et al. 2018 |
| *E.* *chlorostigma* | 6 | epchlB/ FLMNH_I 2006-0681 | 2006 | Indonesia | MK729757 | In this research |
|  |  | epchlC/ FLMNH_I 2007-1089 | 2007 | Indonesia | MK729758 | In this research |
|  |  | JQ412500 | 01-Dec-2009 | New Caledonia | JQ412500 |  |
|  |  | JQ412501 | 01-Dec-2009 | New Caledonia | JQ412501 |  |
|  |  | KM226244 | 23-Jul-2014 | India | KM226244 |  |
|  |  | KM226245 | 23-Jul-2014 | India | KM226245 |  |
| *E.* *tankahkeei* | 9 | epspp/eptan01 | 1-Sep-2011 | Xiamen, Fujian | MF185590 | Qu et al. 2018 |
|  |  | eptan02 | 22-Aug-2016 | Xiamen, Fujian | MK729745 | In this research |
|  |  | eptan03 | 22-Aug-2016 | Xiamen, Fujian | MK729746 | In this research |
|  |  | eptan04 | 15-Feb-2017 | Shenzhen, Guangdong | MK729747 | In this research |
|  |  | eptan05 | 2-Apr-2017 | Shenzhen, Guangdong | MK729748 | In this research |
|  |  | eptan06 | 2-Apr-2017 | Sansha, Hainan | MK729749 | In this research |
|  |  | eptan07 | 1-Jul-2017 | Shenzhen, Guangdong | MK729750 | In this research |
|  |  | eptan08 | 31-Jul-2018 | Xiamen, Fujian | MK729751 | In this research |
|  |  | eptan09 | 22-Mar-2019 | Wenchang, Hainan | MK729752 | In this research |
| *E. gabriellae* | 2 | epgabA | Jan-2018 | Gulf of Oman | MK729759 | In this research |
|  |  | epgabB | Jan-2018 | Oman | MK729760 | In this research |
| *E.* *geoffroyi* | 2 | epgeoA | Mar-2018 | Red Sea | MK729763 | In this research |
|  |  | epgeoB | Mar-2018 | Red Sea | MK729764 | In this research |
| *E.miliaris* | 2 | epmilA/ FLMNH_I 2006-0784 | 2006 | Sulawesi, Indonesia | MK729761 | In this research |
|  |  | epmilB/ FLMNH_I 2006-0728 | 2006 | Indonesia | MK729762 | In this research |
| *E.polylepis* | 4 | eppoyA/ FLMNH_I 2005-1073 | 2005 | India | MK729753 | In this research |
|  |  | eppoyB | Feb-2018 | Dubai | MK729754 | In this research |
|  |  | eppoyC | Jan-2018 | Arabian Sea | MK729755 | In this research |
|  |  | eppoyD | Jan-2018 | Dubai | MK729756 | In this research |
